# Development of a simple treatment eligibility algorithm to decentralise hepatitis B care in Africa

**DOI:** 10.1016/S2468-1253(23)00449-1

**Published:** 2024-02-15

**Authors:** Nicolas Minier, Alice Guingané, Edith Okeke, Edford Sinkala, Asgeir Johannessen, Monique Andersson, Pantong Davwar, Hailemichael Desalegn, Mary Duguru, Fatou Fall, Souleyman Mboup, Tongai Maponga, Philippa C. Matthews, Adria R. Mena, Gibril Ndow, Stian M. S. Orlien, Nicholas Riches, Moussa Seydi, Mark Sonderup, C. Wendy Spearman, Alexander J. Stockdale, Jantjie Taljaard, Michael Vinikoor, Gilles Wandeler, Maud Lemoine, Yusuke Shimakawa, Roger Sombié

**Affiliations:** 1 Insitut Pasteur, Université Paris Cité, Unité d’Épidémiologie des Maladies Émergentes, Paris, France; 2 Hepato-Gastroenterology Department, Bogodogo University Hospital Center, Ouagadougou, Burkina Faso; 3 Faculty of Medical Sciences, University of Jos, Jos, Nigeria; 4 Department of Internal Medicine, University of Zambia, Lusaka, Zambia; 5 Institute of Clinical Medicine, University of Oslo, Oslo, Norway; 6 Department of Infectious Diseases, Vestfold Hospital, Tønsberg, Norway; 7 Nuffield Department of Medicine, University of Oxford, Oxford, United Kingdom; 8 Division of Medical Virology, Stellenbosch University Faculty of Medicine and Health Sciences, Cape Town, South Africa; 9 Medical Department, St. Paul’s Hospital Millennium Medical College, Addis Ababa, Ethiopia; 10 Department of Hepatology and Gastroenterology, Hopital Principal de Dakar, Dakar, Senegal; 11 L’Institut de Recherche en Santé, de Surveillance Épidémiologique et de Formations (IRESSEF), Dakar, Senegal; 12 The Francis Crick Institute, London, United Kingdom; 13 Division of Infection and Immunity, University College London, London, United Kingdom; 14 Institute of Social and Preventive Medicine, University of Bern, Bern, Switzerland; 15 Department of Metabolism, Digestion and Reproduction, Imperial College London, London, United Kingdom; 16 MRC Unit The Gambia, London School of Hygiene & Tropical Medicine, Banjul, Gambia; 17 Department of Clinical Sciences, Liverpool School of Tropical Medicine, Liverpool, United Kingdom; 18 Service de Maladies Infectieuses et Tropicales, Centre Regional de Recherche et de Formation, Centre Hospitalier National Universitaire de Fann, Dakar, Senegal; 19 Division of Hepatology, Department of Medicine, Faculty of Health Sciences, University of Cape Town, Cape Town, South Africa; 20 Department of Clinical Infection, Microbiology and Immunology, Institute of Infection, Veterinary and Ecological Sciences, University of Liverpool, Liverpool, United Kingdom; 21 Malawi-Liverpool-Wellcome Trust Clinical Research Program, Blantyre, Malawi; 22 Division of Infectious Diseases, Department of Medicine, Tygerberg Hospital and Stellenbosch University, Cape Town, South Africa; 23 University of Alabama at Birmingham, Birmingham, Alabama, United States of America; 24 Hepato-Gastroenterology Department, Yalgado Ouédraogo University Hospital Center, Ouagadougou, Burkina Faso

**Keywords:** Hepatitis B virus, sub-Saharan Africa, treatment eligibility, resource-limited settings

## Abstract

**Background:**

To eliminate hepatitis B virus (HBV) infection in resource-limited settings, expanding and decentralizing HBV care services is essential. However, peripheral health facilities often lack access to diagnostic tools necessary for assessing eligibility for antiviral therapy (AVT). Through a multi-regional collaboration in sub-Saharan Africa, we developed and evaluated a simplified algorithm using tests generally available at lower-level health facilities, to evaluate AVT eligibility for people with HBV-infection.

**Methods:**

We first surveyed biomarker availability across different healthcare levels through HEPSANET (Hepatitis B in Africa Collaborative Network). We then divided the largest cross-sectional HBV dataset in sub-Saharan Africa into derivation and validation sets. In the derivation set, we selected a combination of locally-available tests that can best identify individuals meeting the 2017 European Association for the Study of the Liver (EASL) criteria using stepwise logistic regression. In the validation set, we estimated sensitivity and specificity of the simplified algorithms for AVT eligibility.

**Findings:**

Across sites, transaminases (AST, ALT) and platelet counts were generally available at district hospital levels, while hepatitis B e antigen (HBeAg) and point-of-care HBV DNA tests (Xpert) were available at regional/provincial hospital levels or above. Among 2928 treatment-naïve HBV-infected individuals from seven countries, 398 (13·6%) met AVT eligibility per EASL guidelines. The following district-level score was developed: platelet counts (10^9^/L), <100 (+2), 100–149 (+1), ≥150 (±0); AST (IU/L), <40 (±0), 40–79 (+1), ≥80 (+2); and ALT (IU/L), <40 (±0), 40–79 (+1), ≥80 (+2). Using a cut-off of ≥2, the algorithm had a sensitivity of 79% and specificity of 87% to identify treatment-eligible individuals in the validation dataset.

**Interpretation:**

By using platelet counts, AST, and ALT, we can identify the majority of HBV-infected individuals in need of AVT. This implies that clinical staging for HBV can be decentralized to district hospital levels in sub-Saharan Africa.

**Funding:**

None

## Introduction

Globally, hepatitis B virus (HBV) infection is responsible for approximately one million deaths per year, and an estimated 257·5 million people are chronically infected.^1^ In the absence of treatment, about 15–40% of people with chronic HBV infection will experience liver disease progression to cirrhosis, liver failure, or hepatocellular carcinoma (HCC), and an estimated 15–25% will die from HBV-related liver diseases.^2^ In 2016, the World Health Organization (WHO) announced the goal to eliminate viral hepatitis as a public health threats, seeking 90% reduction in new infections and 65% reduction in mortality by 2030.^3^ However, in 2019, it was estimated that only 2% of people chronically infected with HBV globally – and less than 0·1% in sub-Saharan Africa (SSA) – were under treatment,^4^ which calls for rethinking of current treatment strategies.

In order to increase uptake of HBV treatment, it may be important to decentralize the decision to initiate and deliver treatment. Decentralization was a key facilitator in scaling up treatment for human immunodeficiency virus (HIV) in SSA two decades ago, and a similar approach has been suggested for viral hepatitis.^5^ A prerequisite for decentralization is the development of treatment eligibility criteria adapted to the diagnostic tools and type of healthcare workers available in lower-level healthcare facilities. In the case of hepatitis C virus (HCV), growing evidence supported task-shifting of treatment initiation to general practitioners, allowing decentralization to a wider range of healthcare facilities.^6^ Consequently, the WHO now promotes a treat-all approach for HCV, with decentralization down to peripheral health or community-based facilities, and integration with other healthcare services such as tuberculosis, HIV, maternal and child health, and non-communicable diseases.^7^

Meanwhile, the high burden of HBV infection, particularly in resource-limited countries, continues to call for simplified and decentralized models of care.^8^ Today, one of the main obstacles to same-day testing and initiation of anti-HBV treatment is the lack of access to diagnostic tools required by clinical guidelines to identify HBV-infected individuals eligible for treatment.^9^ Indeed, the assessment of treatment eligibility according to international liver societies typically involves costly and sophisticated tests, including HBV DNA quantification, transient elastography (TE), and liver histopathology.^10–12^ However, these tools are rarely available in resource-limited settings, particularly in rural and decentralized contexts, where the majority of people living with hepatitis B reside.^13–16^

In response to the pressing need for a simplified care model that facilitates the decentralization of hepatitis B treatment, we sought to develop simple algorithms tailored to each level of healthcare facility (primary, district, and regional/provincial). Through the Hepatitis B in Africa Collaborative Network (HEPSANET),^17^ we first conducted a questionnaire survey to map the availability of clinical and laboratory parameters at different levels of healthcare facilities in SSA. Second, using the largest cross-sectional dataset of treatment-naïve HBV-infected individuals who underwent comprehensive treatment eligibility assessment in SSA, we identified a minimal combination of locally-available tests that effectively identify HBV-infected individuals meeting the treatment eligibility criteria outlined in the 2017 European Association for the Study of the Liver (EASL) guidelines.^10^

## Material and Methods

### Site survey

We developed a site survey questionnaire by integrating five testing service tiers determined by the WHO hepatitis testing guidelines:^18^ national reference centre (Tier4); provincial/regional hospital (Tier3); district hospital (Tier2); primary care (Tier1); and community/outreach (Tier0). During discussions with HEPSANET investigators, we identified the following clinical and biological parameters that could be valuable in assessing treatment eligibility: sex, age, family history of HCC or cirrhosis, clinical signs of decompensation, platelet count, alanine aminotransferase (ALT), aspartate aminotransferase (AST), gamma-glutamyl transpeptidase (GGT), total bilirubin, prothrombin time, HBeAg, HBV DNA levels, TE, and liver histopathology. In April 2022, we distributed a questionnaire to the principal investigators of all HBV cohorts participating in the HEPSANET consortium and asked to specify the availability and feasibility of each parameter at different levels of healthcare facilities within their respective countries. We determined a set of parameters generally accessible at each healthcare level in SSA based on confirmation of test availability by at least half of the surveyed sites at that specific tier.

### Inclusion and exclusion criteria

We used the cross-sectional database established by the HEPSANET consortium.^17,19^ Briefly, a systematic review was conducted to identify observational studies focusing on individuals with chronic HBV infection in SSA. These studies were at least required to provide data on fibrosis stage and platelet count. Then, authors of these articles were systematically contacted and asked to share anonymized individual data with a comprehensive panel of clinical and biological parameters, including those listed above. All authors agreed to share data, and a total of 13 distinct cohorts from 8 countries participated in this database.^17^ From this database, we included participants meeting the following criteria for this analysis: i) age 18 years or older, ii) have not commenced antiviral therapy; iii) fibrosis stage determined through a valid liver stiffness measurement using transient elastography^20^ or liver histopathology; and iv) having data on ALT, AST, platelet count, and HBV DNA levels. We excluded participants if they met any of the following conditions: presence of metabolic disorders (e.g., diabetes, liver steatosis, hypertension, hyperlipidemia); co-infection with HIV, HCV, hepatitis D virus (HDV), or schistosomiasis; missing data pertaining to age or sex.

### Treatment eligibility

We considered the treatment eligibility by the EASL 2017 guidelines as the reference criteria.^10^ These include fibrosis stage, HBV DNA levels, HBeAg, ALT, first-degree family history of HCC of cirrhosis, and clinical signs of liver decompensations (Supplementary Appendix 1). For those without liver histopathology, significant liver fibrosis and cirrhosis were determined using TE: liver stiffness measurement of ≥7·9 kPa and ≥12·2 kPa, respectively.^19,21,22^ Additionally, we assessed two simplified treatment eligibility criteria: the WHO 2015 guidelines,^23^ and TREAT-B score (Supplementary Appendix 2).^24^

### Derivation and validation datasets

To split the HEPSANET database into the derivation and validation sets, we first arranged the cohorts according to their population size. Then, we assigned each cohort, starting with the derivation dataset and then to the dataset with fewer participants. This ensured that the relative sizes of the datasets were closely matched and limited the chances of overfitting the algorithm to the derivation set.

### Statistical analysis

We reported participant characteristics for the derivation and validation sets. Differences between the groups were assessed using Chi-square tests for categorical variables and Student’s t-tests or Wilcoxon rank-sum test for continuous variables. We considered p<0·05 as statistically significant. Factors associated with the EASL eligibility criteria were assessed using univariable logistic regression, with log-transformed biological marker levels.

We developed tier-specific decision trees for treatment eligibility based on available parameters at each tier level. The primary criteria included “family history of HCC or cirrhosis” or “clinical diagnosis of decompensated cirrhosis”, immediately triggering eligibility independent of other parameters. For individuals not meeting these decisive criteria, we built tier-specific multivariable regression models using parameters available at each tier. We did not consider parameters missing for more than 20% of participants in the database. Model selection employed backward stepwise selection with entry and removal p-values set at 0·01 and 0·005, respectively, determined by Wald tests. To simplify the algorithm, we transformed the multivariable logistic regression model into a point-based scoring system, following a methodology described by Sullivan and colleagues.^25^

Diagnostic performance of the tier-specific simplified algorithms was assessed separately in both the derivation and validation sets, with reference to the EASL 2017 criteria. The capability to discriminate between eligible and non-eligible individuals was evaluated using receiver operating characteristic (ROC) curves and the optimal score cut-off was chosen to maximize Youden’s J statistic within the derivation dataset. We compared the area under the ROC curve (AUROC) of the new algorithms to that of the WHO 2015 and TREAT-B. We conducted subgroup analyses based on sex, age, excessive alcohol intake, screening venue, and region. All analyses were performed using STATA 17 (Stata Corporation, USA).

## Results

### Availability of clinical and laboratory parameters

Of 13 distinct cohorts from eight countries, data regarding the availability of parameters at different levels of healthcare facilities were provided by eleven cohorts: Burkina Faso (n=2), Ethiopia (n=1), The Gambia (n=1), Malawi (n=1), Nigeria (n=1), Senegal (n=2), South Africa (n=2), and Zambia (n=1). The majority of sites reported that parameters such as sex, age, family history, and clinical diagnosis of jaundice could be accurately determined at the primary-health level (Tier1), as reported in Table 1. Haematology and biochemistry tests, including platelet counts, ALT, AST, GGT, bilirubin, and prothrombin time were available in most sites starting from the district hospital level or higher (Tier2). In addition, HBeAg test (both rapid diagnostic test (RDT) and laboratory-based immunoassays) and point-of-care (PoC) HBV DNA assay (Xpert®, Cephaid, US) were available in more than half of the sites from the regional/provincial hospital level (Tier3). Finally, laboratory platforms for HBV DNA quantification, TE, and liver histopathology were predominantly limited to the national hospital level (Tier4). These results indicated that the applicability of the EASL 2017 guidelines, based on HBV DNA assay and TE or liver histopathology, is primarily limited to the national hospital level, while the WHO 2015 guidelines and the TREAT-B score can be used at the regional/provincial hospital level. None of these existing eligibility criteria could be effectively applied at the district hospital level or below.

### Study population

**Figure 1:**
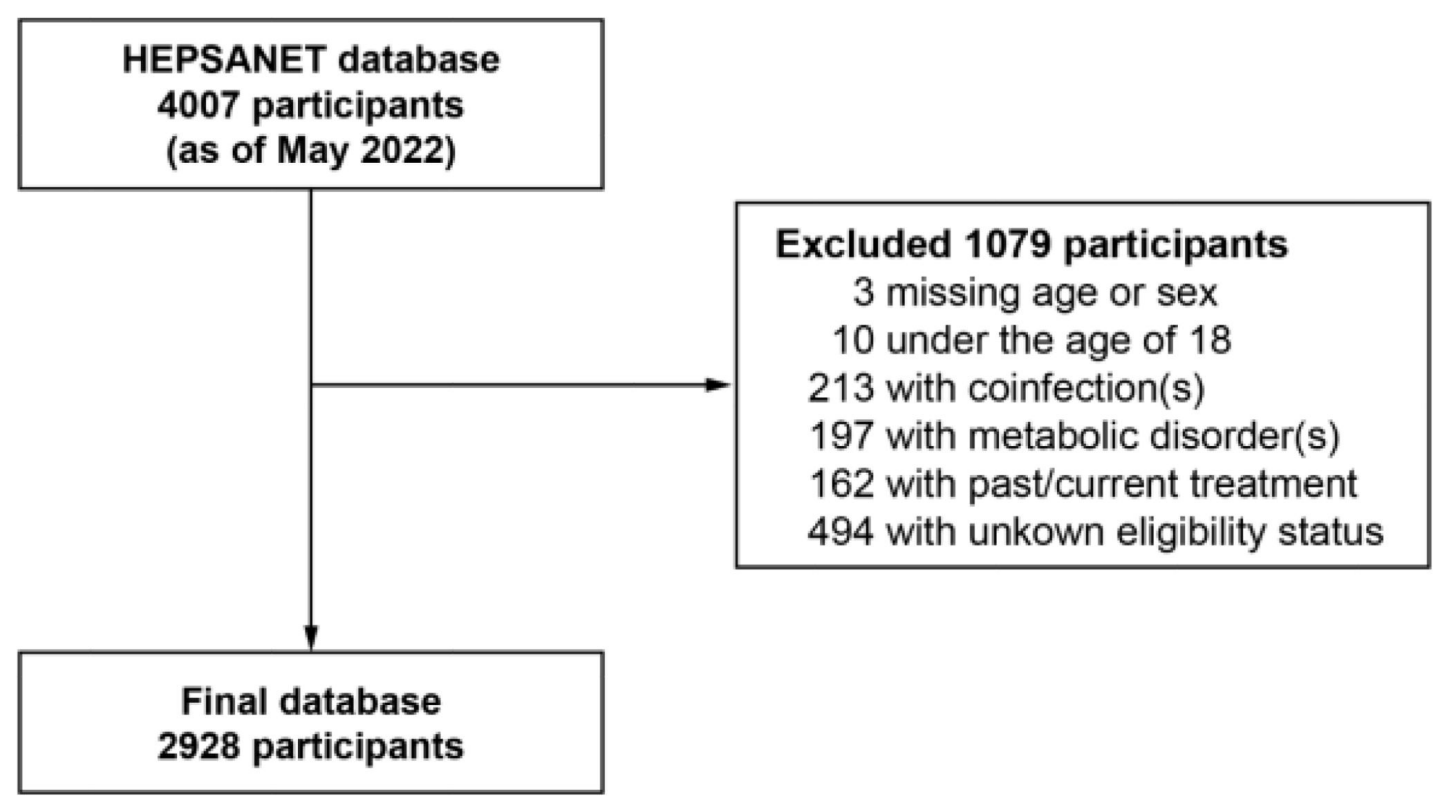
Flowchart of participant inclusion

By applying the inclusion criteria for the current analysis, a total of 2928 participants from 12 cohorts representing seven African countries were included in this analysis (Figure 1): Ethiopia (n=945); The Gambia (n=783), Senegal (n=764), Burkina Faso (n=134), South Africa (n=129), Malawi (n=91), and Zambia (n=82). The geographic origin of these cohorts is presented in Supplementary Appendix 3.

Table 2 summarizes the characteristics of the study participants. The majority were men (60·3%), and the average age was 35 years (SD ± 11). The proportion eligible for treatment was 13·6%, 8·3%, 23·6%, using the EASL 2017 guidelines, the WHO 2015 guidelines, and the TREAT-B, respectively. The allocation of cohorts to derivation (n=1476) and validation datasets (n=1452) according to the population size resulted in statistically significant differences in the characteristics of these two groups. Overall, the derivation set had a younger average age (34±10 vs 37±11) and a lower proportion of men (56·2% vs 64·5%). Furthermore, significant differences were observed in the distribution of laboratory parameters, as well as in the proportion of participants eligible for treatment according to the EASL 2017 guidelines (Table 2).

### Development of the new algorithms

Using the whole dataset (N=2,928), the univariable analysis identified the following candidate parameters to be significantly associated with the EASL 2017 treatment eligibility: male sex; positive HBeAg; increased levels of ALT, AST, GGT, bilirubin, and prothrombin time; and decreased levels of platelet count (Supplementary Appendix 4). Given the large number of participants with missing items (>20%), bilirubin and prothrombin time were not considered in subsequent analyses.

Using the derivation set, we developed tier-specific simplified algorithms. Of the parameters available at the primary-health level (Table 1), the presence of family history of cirrhosis/HCC or clinical diagnosis of jaundice immediately identified 25·2% (63/250) of individuals eligible for the EASL criteria in the derivation set. For the remaining individuals in this population, we constructed a multivariable model by incorporating age and sex, which were the only two additional parameters available at this tier. The stepwise procedure identified sex as the sole predictor of eligibility (Supplementary Appendix 6).

**Figure 2:**
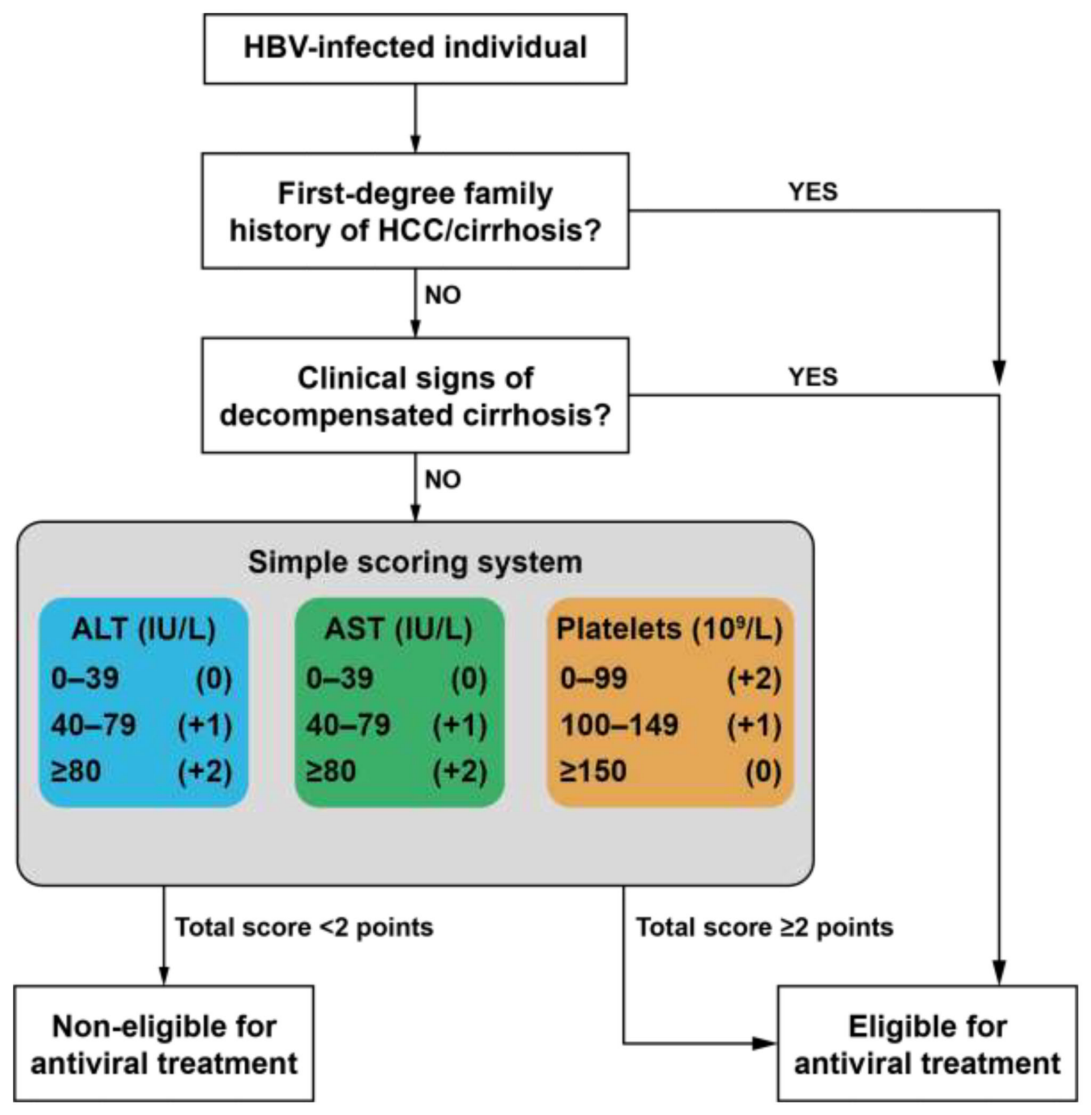
HEPSANET algorithm enabling to assess treatment eligibility at the district hospital level (Tier 2) in sub-Saharan Africa Abbreviations: alanine aminotransferase (ALT), aspartate aminotransferase (AST), hepatocellular carcinoma (HCC)

At the district hospital level, the presence of family history or clinical diagnosis of decompensated cirrhosis (including jaundice, ascites, encephalopathy, and variceal bleeding) identified 56·8% (142/250) of individuals eligible by EASL criteria. For the remaining individuals, we developed a multivariable model by considering age, sex, platelet count, AST, ALT, and GGT, which were the parameters available at this level. The logistic regression model that emerged, after stepwise selection of predictors, was as below: risk score = - 0·7960 + [1·2232 × ln(ALT)] + [0·7939 x ln(AST)] - [1·4674 x ln(platelets)], where ALT and AST levels were expressed in IU/L and platelet count was in 10^9^/L. These regression coefficients were subsequently converted into integer points (Supplementary Appendix 5). The total point for this score was obtained by summing the scores for ALT (<40 IU/L, 0 point; 40–79 IU/L, 1 point; ≥80 IU/L, 2 points), AST (<40 IU/L, 0 point; 40–79 IU/L, 1 point; ≥80 IU/L, 2 points), and platelet count (<100 x10^9^/L, 2 points; 100–149 x10^9^/L, 1 point; ≥150 x10^9^/L, 0 point). Figure 2 provides an illustrative presentation of the district-level algorithm.

Finally, at the regional/provincial level, 142 individuals were excluded from the derivation set, since they were identified by family history or clinical diagnosis of liver decompensation. The multivariable logistic regression, fed by HBeAg in addition to Tier2 point score, produced an algorithm similar to that of Tier2, which considered HBeAg score (positive, 2 points; negative, 0 point) in addition to the one developed for the district hospital level (Supplementary Appendix 6).

### Performance of the new algorithms

Using the derivation set, the optimal cut-offs for each score were determined to maximize the sum of sensitivity and specificity: ≥2 points for the district-level score and ≥2 points for the regional/provincial-level score. By applying these cut-offs, we calculated the sensitivity and specificity of each algorithm to identify individuals eligible for antiviral therapy according to the EASL guidelines in both the derivation and validation datasets.

In the derivation dataset, the AUROC, sensitivity, and specificity were 0·68 (95% CI: 0·66–0·71), 90%, 47% for the primary-level algorithm, 0·88 (95% CI: 0·86–0·91), 82%, 94% for the district-level algorithm, and 0·91 (95% CI: 0·89–0·93), 92%, 89% for the regional/provincial-level algorithm (Table 3).

In the validation dataset, the AUROC, sensitivity, and specificity were 0·63 (95% CI: 0·60–0·65), 89%, 37% for the primary-level algorithm, 0·83 (95% CI: 0·79–0·86), 79%, 87% for the district-level algorithm, and 0·88 (95% CI: 0·85–0·90), 91%, 84% for the regional/provincial-level algorithm (Table 3).

We also evaluated the performance of the existing simplified criteria (WHO 2015 and TREAT-B). In the validation set, WHO 2015 had higher AUROC than the primary-level algorithm (0·77, 95% CI: 0·73–0-80, versus 0·68, 95% CI: 0·66–0·77, p=0·02), but lower than that of other newly developed algorithms. In the entire cohort, the sensitivity and specificity of WHO 2015 were 48% and 98%, respectively. In contrast, TREAT-B had AUROC comparable to our regional/provincial-level algorithms (0·88, 95% CI: 0·86–0-91, versus 0·88, 95% CI: 0·66–0·91). The sensitivity and specificity of TREAT-B were 90% and 86%, respectively.

### Subgroup analysis

Subgroup analyses for the performance of newly developed algorithms in the validation set were presented in Supplementary Appendix 7. The performance of the district-level algorithm, represented by AUROC, did not vary according to sex, age, excessive alcohol intake, sub-region (West versus East/South), or screening venue (hospital-based versus community-based). For the regional/provincial-level algorithm, however, the inclusion of HBeAg into the scoring system led to varying performance in certain groups: <30 years (AUROC: 0·82, 95% CI: 0·77–0·88) versus ≥30 years (AUROC: 0·90, 95% CI: 0·87–0·93, p=0·01); West Africa (AUROC: 0·88, 95% CI: 0·86–0·91) versus East/South Africa (AUROC: 0·77, 95% CI: 0·68–0·87, p=0·04); and hospital-based screening (AUROC: 0·86, 95% CI: 0·82–0·89) versus community-based (AUROC: 0·91, 95% CI: 0·87–0·94, p=0·05).

## Discussion

In our site survey conducted across eight countries in SSA, we have confirmed that the conventional treatment eligibility criteria for hepatitis B infection, which rely on HBV DNA PCR and TE, prove to be impractical at lower-level healthcare facilities, such as district hospitals. This represents a key barrier to successful implementation of decentralised HBV care under current guidelines. In contrast, through the integration of readily available parameters at the district hospital level - both clinical (family history of HCC or cirrhosis and clinical diagnosis of decompensated cirrhosis) and laboratory (ALT, AST, and platelet counts) - we were able to accurately identify 79% of individuals eligible for treatment and 87% of those ineligible for treatment. These findings emphasize the feasibility of decentralizing clinical staging in SSA, even without upgrading laboratory infrastructure.

Various approaches exist for simplifying HBV treatment criteria. HEPSANET score, for instance, was developed using a purely statistical methodology. From a range of clinical and biological markers readily available at different levels of healthcare facilities in SSA, an automated stepwise selection procedure was employed to identify a subset of variables that exhibited a robust predictive capacity for determining treatment eligibility. An alternative approach involves substituting each of the conventional reference tests used to ascertain treatment eligibility with inexpensive and accessible alternatives. For example, the assessment of liver fibrosis can be conducted using the AST-to-platelet ratio index (APRI) or GGT-to-platelet ratio (GPR) in lieu of more resource-intensive methods.^19,26^ Instead of quantifying HBV DNA levels using real-time PCR, the detection of high HBV viral replication can be accomplished through a rapid diagnostic test, such as hepatitis B core-related antigen (HBcrAg).^26^ Further studies are needed to determine which simplified approach better identifies individuals in need of treatment.

Importantly, the laboratory tests required for the district-level algorithm (AST, ALT, and platelet count) are all included in the WHO’s list of essential in-vitro diagnostic tests for clinical laboratories.^27^ By incorporating HBeAg into these three tests, our regional/provincial-level algorithm substantially improved sensitivity, increasing from 79% to 91%, while maintaining a similar degree of specificity (87% in the district-level algorithm and 84% in the regional/provincial-level algorithm). Indeed, HBeAg test is also included in the WHO’s list of essential tests, with HBeAg RDT for health facilities without laboratory and HBeAg immunoassay, such as enzyme-linked immunoassay (ELISA) for clinical laboratories.^27^ However, an important limitation of an algorithm utilizing HBeAg is the limited availability of laboratory-based HBeAg tests in SSA (Table 1) and the lack of analytical sensitivity in HBeAg RDTs.^28,29^ In our HEPSANET dataset, HBeAg was assessed using laboratory-based immunoassays; therefore, the performance of the regional/provincial-level algorithm based on HBeAg RDT might be less accurate. This highlights the urgent, unmet need to improve the analytical sensitivity of HBeAg RDTs.

Our study has also highlighted an important finding: even in primary healthcare facilities without laboratories, 89% of individuals in need of antiviral therapy can be identified using a simple algorithm based on family history of HCC/cirrhosis, clinical diagnosis of jaundice, and in the absence of these histories or clinical signs, proposing treatment for all men. Nevertheless, the specificity was only 37%, indicating that a majority of ineligible individuals would receive potentially unnecessary treatment. Given that the current antiviral therapy regimen often requires lifelong treatment, and individuals in SSA often have to pay out of pocket in the absence of national subsidization, there is a debate about whether it is justifiable to treat a large portion of the population for whom benefits are less well established, and therefore do not meet current eligibility criteria.

Some shortcomings must be noted in our study. Firstly, the reference standard used to develop and evaluate the simplified algorithm was eligibility for treatment based on the EASL guidelines, which may not be a perfect predictor of adverse outcomes in SSA. Ideally, a longitudinal study following a well-characterized cohort of untreated HBV-infected individuals and identifying a combination of baseline factors predictive of hard endpoints like cirrhosis or HCC would provide the most informative results. However, such comprehensive data are scarce in SSA, with only a few studies available, and this is a research priority for HEPSANET.^30^ Secondly, due to our reliance on cross-sectional data, we were unable to assess the impact of fluctuations in transaminase levels or other biomarkers over time. Thirdly, the site survey was conducted through the investigators participating in the HEPSANET, which has a bias toward research-active and tertiary centres, rather than involving systematic sampling of provinces or countries within the continent. This was done for practical reasons, and could raise the question of the generalizability of the site survey findings to other populations in SSA. However, we note that our results, targeting parameters of interest to our specific study, are consistent with the overall landscape pictured by previous studies that attempted to describe availability of various testing and diagnoses across countries and levels of healthcare facilities.^14,16^

Our study also had certain strengths, mainly the representation of multiple cohorts from seven countries, spanning eastern, western, and southern Africa. The dataset was meticulously constructed through a systematic review, ensuring that no relevant cohorts were omitted from our analysis. Second, the large sample size allowed us to develop and evaluate the algorithms using independent derivation and validation datasets, thus avoiding overestimation of the performance of the new scoring system. Third, the resource availability questionnaire allowed us to propose biomarkers that are available and relevant to the end-users in SSA. With these robust considerations of resource availability, we believe that the proposed scoring system could be used to facilitate decentralization of hepatitis care.

In conclusion, we developed a new and simple algorithm – based on transaminases and platelets – to aid the decision to initiate antiviral treatment for HBV-infected patients in SSA. This can help countries decentralize HBV therapy from national reference centers to district hospitals, allowing for task sharing to non-specialist doctors or nurses with access to only basic laboratory support. The simple scoring system reported here could be translated to policy and help countries in SSA achieve the goal of eliminating viral hepatitis as a public health threat by 2030.

## Supplementary Material

Supplementary Appendix

## Figures and Tables

**Table 1:** Availability of clinical and laboratory parameters at different levels of healthcare facilities in sub-Saharan Africa

Parameters	Tiered level of healthcare facility*					Tier considered for analysis
	Tier0	Tier1	Tier2	Tier3	Tier4	
**Clinical parameters**						
**Sex**	100%	100%	100%	100%	100%	0
**Age**	100%	100%	100%	100%	100%	0
**1st degree family history (HCC, cirrhosis)**	73%	82%	100%	100%	100%	0
**Clinical diagnosis of jaundice**	73%	82%	100%	100%	100%	0
**Clinical diagnosis of ascites**	27%	45%	100%	100%	100%	2
**Clinical diagnosis of hepatic encephalopathy**	27%	36%	82%	100%	100%	2
**Clinical diagnosing of variceal bleeding**	18%	18%	55%	82%	100%	2
**Laboratory parameters**						
**Full blood count (platelets)**	9%	36%	100%	100%	100%	2
**Alanine aminotransferase (ALT)**	9%	36%	91%	91%	100%	2
**Aspartate aminotransferase (AST)**	9%	36%	91%	91%	100%	2
**Gamma-glutamyl transferase (GGT)**	0%	9%	55%	73%	100%	2
**Bilirubin**	0%	18%	64%	73%	100%	2
**Prothrombin time (INR)**	0%	18%	55%	64%	73%	2
**HBeAg (Rapid Diagnosis Test)**	9%	18%	36%	60%	55%	3
**HBeAg (Laboratory-based immunoassays)**	0%	0%	18%	45%	82%	3
**HBV DNA (Xpert)**	0%	0%	9%	55%	82%	3
**HBV DNA (Conventional platform)**	0%	0%	9%	27%	73%	4
**Transient elastography (Fibroscan)**	0%	0%	0%	9%	82%	4
**Liver biopsy**	0%	0%	0%	9%	100%	4
**Histopathology**	0%	0%	0%	9%	82%	4

* WHO tiered levels of healthcare facilities: Tier0, community/outreach; Tier1, primary care; Tier2, district hospital; Tier3, provincial/regional hospital; Tier4, national reference centre.

**Table 2:** Characteristics of study participants (N=2,928)

Variables	No. with missing information	Whole cohort	Derivation set	Validation set	p-value
***Clinical variables***					
Male		1765 (60·3)	829 (56·2)	936 (64·5)	<0·001
Age (years)		35±11	34±10	37±11	<0·001
≥30		1960 (66·9)	886 (60·0)	1074 (74·0)	<0·001
BMI (kg/m2)	n=400	22·9±4·8	22·7±5·1	23·1±4·5	0·013
≥30		166 (6·6)	75 (6·2)	91 (6·9)	0·442
Family history of HCC	n=779	84 (3·9)	46 (4·0)	38 (3·8)	0·722
Excessive alcohol intake	n=719	164 (7·4)	45 (3·7)	119 (12·0)	<0·001
***Clinical signs of decompensation***					
Ascites		86 (2·9)	83 (5·6)	3 (0·2)	<0·001
Variceal bleeding		21 (0·7)	21 (1·4)	0 (0·0)	<0·001
Jaundice		19 (0·6)	17 (1·2)	2 (0·1)	0·001
Hepatic encephalopathy		0 (0·0)	0 (0·0)	0 (0·0)	----------
***Hepatitis B virus markers***					
HBeAg	n=88	237 (8·8)	126 (10·0)	111 (7·7)	0·036
Viral load (log10 IU/mL)		2·88±1·6	2·98±1·5	2·75±1·7	<0·001
***Biochemistry and hematology***					
ALT (IU/L)		31±31	29±26	33±35	<0·001
AST (IU/L)		34±36	33±39	36±33	<0·001
GGT (IU/L)	n=584	35±46	35±56	34±34	<0·001
Bilirubin (mg/dL)	n=796	0·80±1·4	0·88±1·9	0·71±0·7	0·04
Platelets (10^9^/L)		238±85	269±88	207±70	<0·001
INR	n=2803	1·12±0·2	1·12±0·2	----------	----------
APRI		0·48±1·1	0·40±0·7	0·56±1·4	<0·001
FIB-4		1·17±1·7	0·97±1·4	1·37±1·9	<0·001
***Elastography***					
Liver stiffness (kPa)	n=41	7·5±8·7	8·6±11	6·4±4·8	0·028
***Liver histopathology***	n=2802				0·889
METAVIR F0		27 (21·4)	2 (20·0)	25 (21·6)	
METAVIR F1		58 (46·0)	6 (60·0)	52 (44·8)	
METAVIR F2		16 (12·7)	1 (10·0)	15 (12·9)	
METAVIR F3		14 (11·1)	0 (0·0)	14 (12·7)	
METAVIR F4		11 (8·7)	1 (10·0)	10 (8·6)	
***Treatment eligibility***					
EASL 2017		398 (13·6)	250 (16·9)	148 (10·2)	<0·001
WHO 2015		243 (8·3)	159 (10·8)	84 (5·8)	<0·001
TREAT-B		690 (23·6)	372 (25·2)	318 (21·9)	<0·001

ALT: alanine aminotransferase. APRI: AST to platelet ratio index. AST: aspartate aminotransferase. BMI: body mass index. GGT: gamma-glutamyl transpeptidase. HBeAg: hepatitis B e antigen. HCC: hepatocellular carcinoma. INR: international normalized ratio.

**Table 3:** Performance of the tier-specific HEPSANET algorithms to identify people eligible for antiviral therapy according to the EASL 2017 guidelines

Test	Number	AUROC [95% CI]	Se (%)	Sp (%)	TP	FN	FP	TN
**In the derivation dataset**	n=1476							
***HEPSANET algorithms***								
Tier1	n=1476	0·68 [0·66–0·71]	90	47	224	26	647	579
Tier2	n=1476	0·88 [0·86–0·91]	82	94	206	44	71	1155
Tier3	n=1258	0·91 [0·89–0·93]	92	89	217	19	109	913
***WHO 2015 guidelines***	n=1476	0·77 [0·73–0·80]	55	98	137	113	22	1204
***TREAT-B***	n=1263	0·88 [0·86–0·91]	90	86	218	23	140	882
**In the validation dataset**	n=1452							
***HEPSANET algorithms***								
Tier1	n=1452	0·63 [0·60–0·65]	89	37	131	17	827	477
Tier2	n=1452	0·83 [0·79–0·86]	79	87	117	31	174	1130
Tier3	n=1438	0·88 [0·85–0·90]	91	84	132	13	202	1091
***WHO 2015 guideline***	n=1452	0·68 [0·64–0·72]	38	98	56	92	28	1276
***TREAT-B***	n=1439	0·87 [0·84–0·90]	88	86	129	17	185	1108

**Se**: sensitivity. **Sp**: specificity. **TP**: true positives. **FN**: false negatives. **FP**: false positives. **TN**: true negatives.

## Data Availability

The data supporting our findings are available from the corresponding author upon reasonable request. The code (STATA) used for this analysis is available from GitHub (https://gitlab.com/NicolasMINIER/hepsanet_simplified_hbv_treatment_eligibility_in_ssa).

## Research In Context

### Evidence before this study

WHO Africa is a region of high hepatitis B virus (HBV) endemicity. While antiviral therapy (AVT) has become increasingly accessible and affordable in this region, only a small fraction of eligible individuals (0·1% in 2019, as estimated by WHO) actually receive these treatments. This disparity may be partly attributed to lack of access to diagnostic tools in decentralized healthcare facilities to determine AVT eligibility, including HBV DNA testing, transient elastography, and liver histopathology. To identify alternative eligibility criteria based on locally-available tests in resource-limited decentralized settings, we searched PubMed and Scopus for articles published until August 2023 using the terms “hepatitis B” AND “treatment eligibility” AND (“simplification” OR “decentralization”). This search identified only two alternative algorithms designed to simplify treatment eligibility assessment. The first, known as the TREAT-B score, relies on alanine transferase (ALT) levels and hepatitis B e antigen (HBeAg). This score was developed in a West African HBV cohort, and has been later evaluated in 11 external cohorts in sub-Saharan Africa, Southeast Asia, Australia, and Europe. Overall, the TREAT-B yielded a median sensitivity of 74% (range: 43-96%) and a median specificity of 77% (range: 41-98%) to identify HBV-infected individuals eligible for AVT. The second, more recent algorithm, HePAA score was developed in a Thai cohort, and incorporated platelet count and albumin levels, in addition to ALT and HBeAg. While these simplified scoring systems might be useful alternative, both require HBeAg testing. Currently, accurate HBeAg detection requires laboratory-based immunoassays, which are not widely available in decentralized healthcare facilities across sub-Saharan Africa.

### Added value of this study

Through the Hepatitis B in Africa Collaborative Network (HEPSANET), we first conducted surveys at ten sites in sub-Saharan Africa. We found that transaminases (AST, ALT) and platelet counts were generally accessible at the district hospitals, while HBeAg and near point-of-care HBV DNA tests (Xpert) were available at regional/provincial hospitals or above, and transient elastography and conventional quantitative HBV DNA tests were limited to national hospitals. Consequently, we developed a simplified algorithm based on the tests available at the district hospital level. We divided the largest cross-sectional HBV dataset in sub-Saharan Africa into derivation and validation sets. As the reference, we considered the 2017 European Association for the Study of the Liver (EASL) eligibility criteria, which rely on conventional tests including HBV DNA testing and transient elastography or liver biopsy. Using stepwise logistic regression, we identified AST, ALT, and platelet count as the best combination for identifying individuals meeting the EASL criteria at the district hospital-level in the derivation set. In the validation set, the algorithm had a sensitivity of 79% and specificity of 87% to identify treatment-eligible individuals.

### Implications of all the available evidence

Our study demonstrates that by utilizing basic tests commonly available at district hospital levels, we can identify the majority of HBV-infected individuals in need of antiviral therapy. This tool has the potential to support the expansion of hepatitis B elimination initiatives in sub-Saharan Africa by enabling decentralized hepatitis B care, which will be critical to reduce cirrhosis and hepatocellular carcinoma in rural communities.

## Data Sharing

De-identified participant data will be shared upon reasonable request after approval by the scientific committee of the research group.
