# Supplementary material for: Development of a simple treatment eligibility algorithm to decentralise hepatitis B care in Africa
Source: Lancet Gastroenterol Hepatol. Author manuscript; Available in PMC 2024 May 29. (PMC7616035; doi:10.1016/S2468-1253(23)00449-1)
Supplement: Supplementary Appendix [file EMS196195-supplement-Supplementary_Appendix.pdf]

Supplementary Appendix 1: Eligibility criteria of EASL 2017 guidelines.

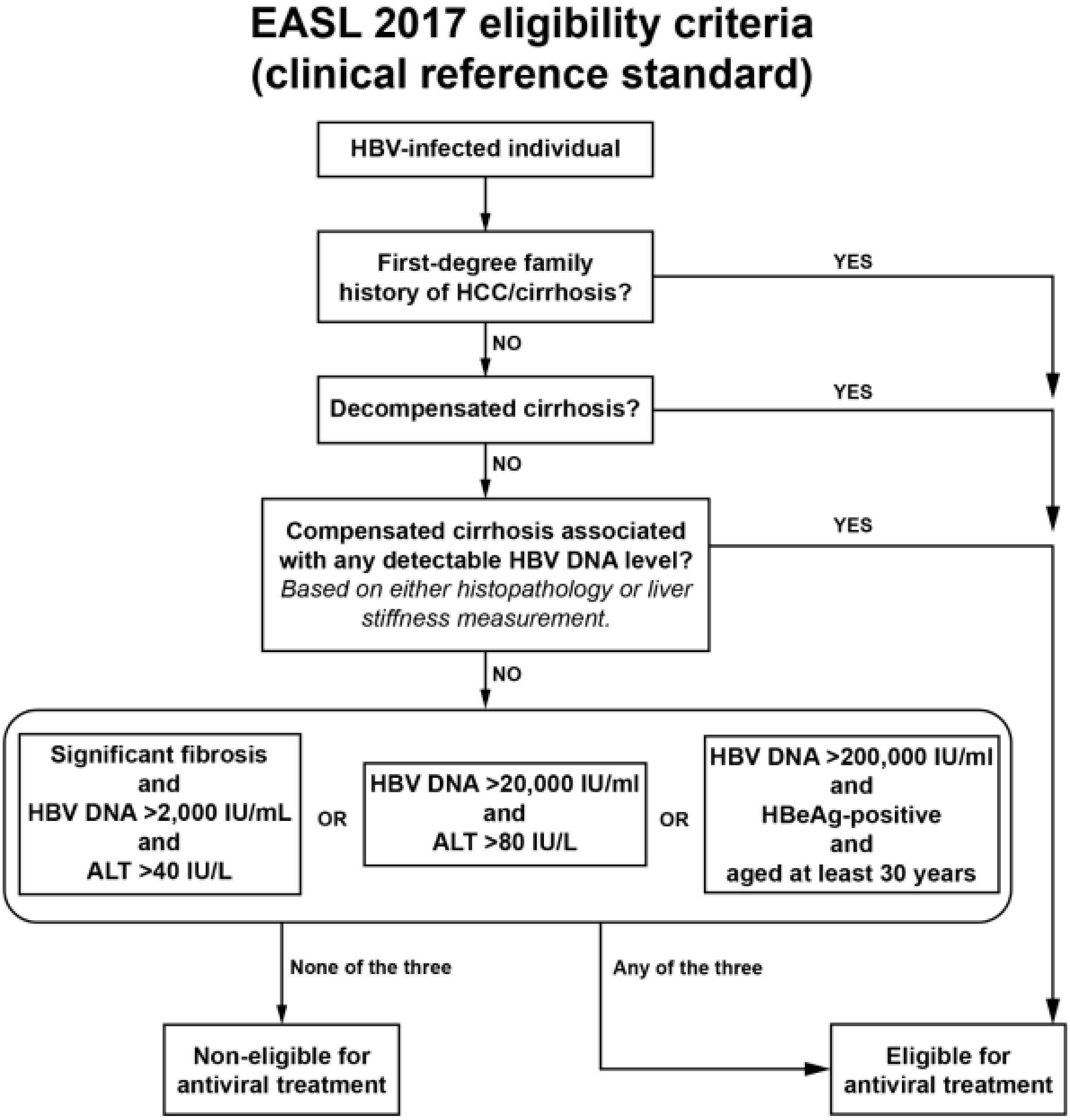

Please note that these algorithms correspond only to the transcription of more complete guidelines into decision algorithms for eligibility to treatment for the purpose of our analysis.

Supplementary Appendix 2: Eligibility criteria of two comparative algorithms. WHO 2015 guidelines, and TREAT-B decision algorithm.

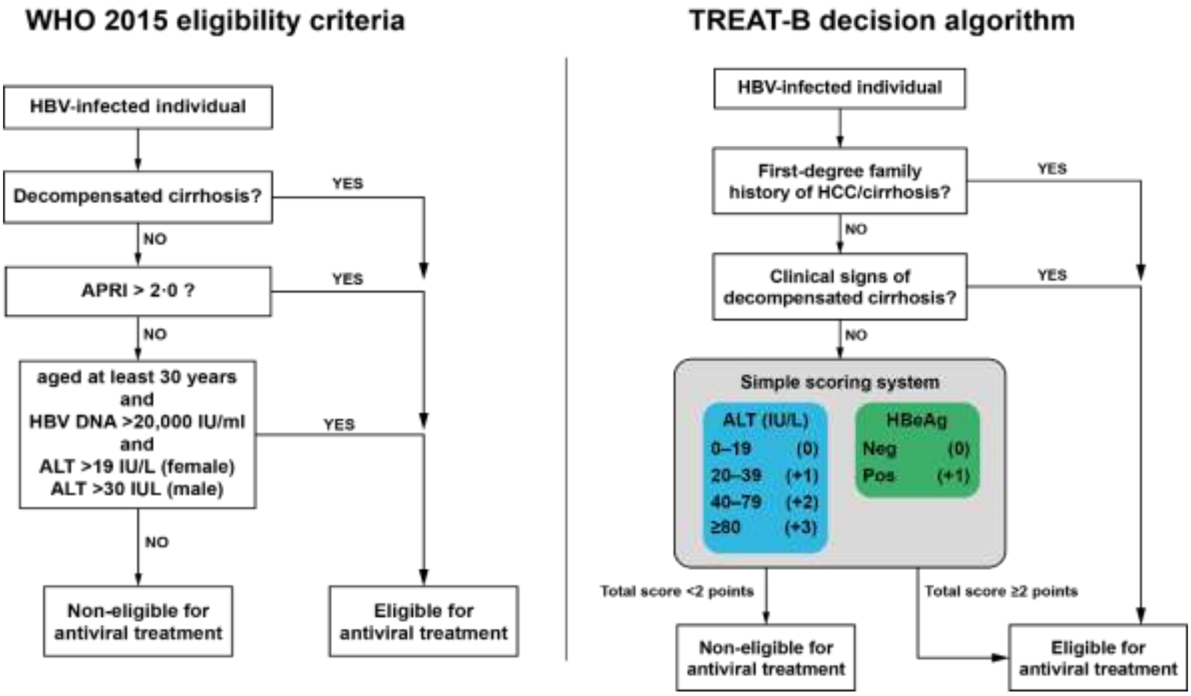

Please note that these algorithms correspond only to the transcription of more complete guidelines into decision algorithms for eligibility to treatment for the purpose of our analysis.

Supplementary Appendix 3: Geographic origin of included cohorts in the derivation and validation sets

Cohort	Size	Derivation set	Validation set
Addis Ababa, Ethiopia (A. Johannessen *et al.*, 2017-2022)	945	945	0
Gambia (PROLIFICA, 2016-2020)	783		783
Thies, Senegal (Sow *et al.*, 2022)	300		1083
Dakar, Senegal (Seydi & Wandeler, unpublished)	234	1179	
Senegal (PROLIFICA, 2016)	153		1236
Cape Town, South Africa (Spearman & Sonderup, unpublished)	105	1284	
Ouagadougou, Burkina Faso (Alice Guingané *et al.*, in writing)	102		1338
Blantyre, Malawi (A. Stockdale *et al.*, 2022)	91	1375	
Lusaka, Zambia (Vinikoor *et al.*, 2018-2020)	82		1420
Dakar, Senegal (Hopital Principal de Dakar, 2011-2018)	77	1452	
Burfina Faso (PROLIFICA, 2019)	32		1452
Cape Town, South Africa (Maponga *et al.*, 2018-2020)	24	**1476**	**1452**

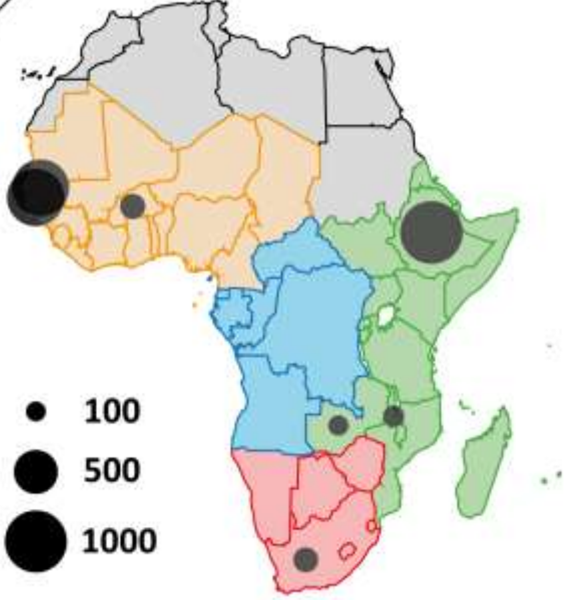

Supplementary appendix 4: Clinical and laboratory parameters associated with the treatment eligibility determined by the EASL 2017 guidelines (N=2,928)

Variable	No. with missing information	EASL non-eligible (n=2530)	EASL eligible (n=398)	p-value
**Male sex**		1474 (58·3%)	291 (73·1%)	<0·001
**Age (years)**		35±11 (33)	35±10 (33)	0·361
**≥30**		1688 (66·7%)	272 (68·3%)	0·522
**HBeAg**		92 (4·0%)	145 (38·1%)	<0·001
**ALT (IU/L)**		27±18 (23)	58±64 (39)	<0·001
**AST (IU/L)**		29±21 (26)	64±75 (41)	<0·001
**Platelets (10^9/L)**		244±82 (237)	201±96 (186)	<0·001
**GGT (IU/L)**	**n=584**	30±29 (23)	69±96 (40)	<0·001
**Bilirubin (mg/dL)**	**n=796**	0·7±0·9 (0·6)	1·4±3·1 (0·7)	<0·001
**INR**	**n=2803**	1·1±0·1 (1·1)	1·3±0·4 (1·1)	0·002

Values are reported as mean±standard deviation (median), or n (percentage).

Supplementary Appendix 5: Simplified scoring system enabling to assess treatment eligibility at the district hospital level

Parameter	Category	Reference	β	Regression distance *Δref.* * β	Points
Scoring system available in Tier2 (district hospital level)					
Platelets (10^9/L)	≥150	5·298	-1·467	0 (base category)	0
	100–149	4·828		0·690	1
	<100	4·437		1·264	2
AST (IU/L)	<40	3·239	0·794	0 (base category)	0
	40–79	4·094		0·679	1
	≥80	4·883		1·305	2
ALT (IU/L)	<40	3·157	1·223	0 (base category)	0
	40–79	4·094		1·147	1
	≥80	4·771		1·974	2

Multivariable regression analysis enabled the quantification of the risk increase or decrease associated with changes in the parameters' values. Normalization using a constant facilitated the generation of distances within a practical and usable range. Rounding the normalized distances resulted in a simplified point-based system.

NB: the reference is expressed as the natural logarithm of the continuous variables.

Supplementary Appendix 6: Tier-specific HEPSANET algorithms to identify people eligible for antiviral therapy in sub-Saharan Africa*

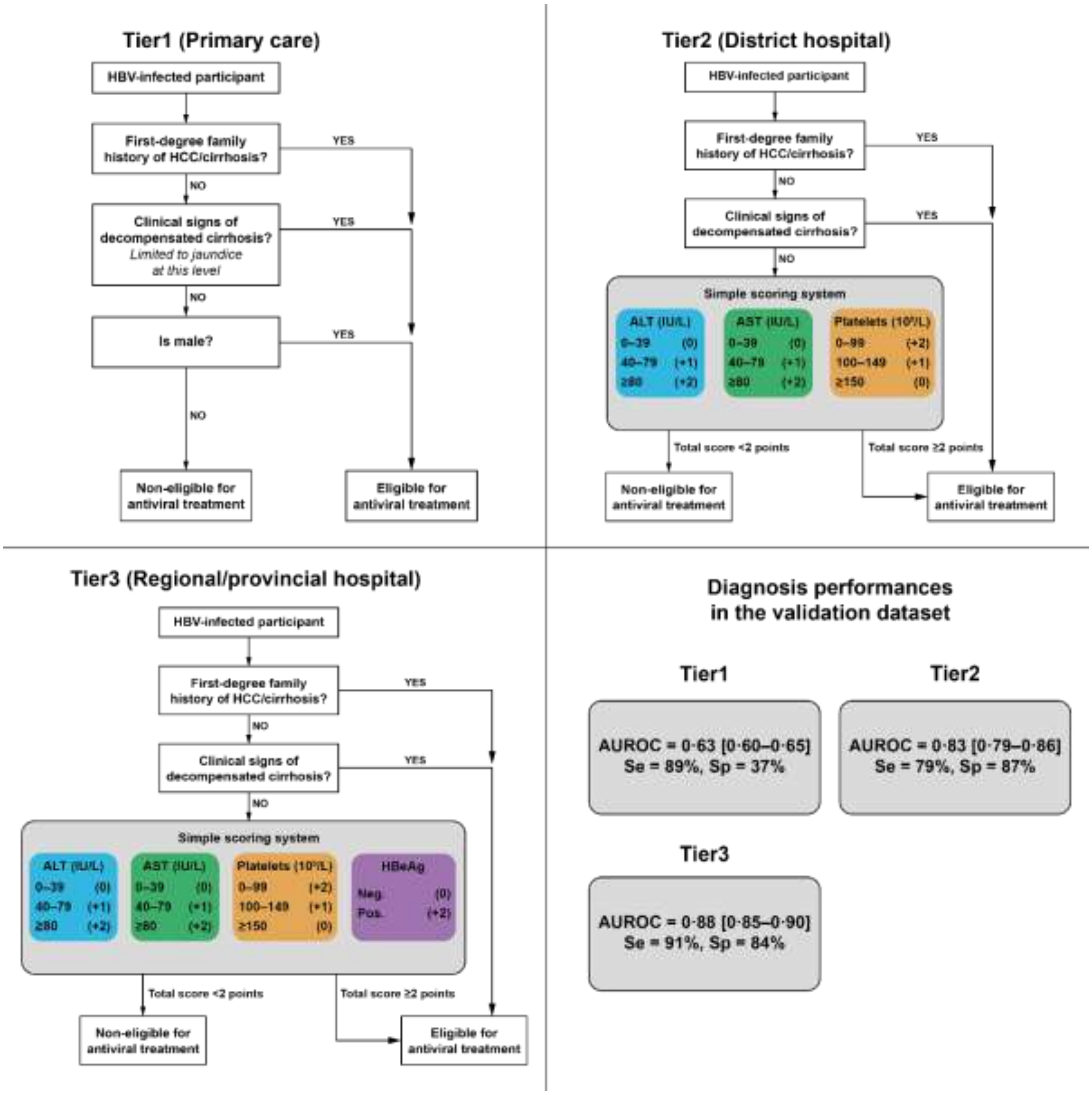

AUROC, sensitivity (Se) and specificity (Sp) reported here correspond to that measured in the validation dataset.

Supplementary Appendix 7: Subgroup analysis for the performance of tier-specific HEPSANET algorithms to identify people eligible for antiviral therapy according to the EASL 2017 guidelines (derivation dataset: n=1,452)

Test	n	(%)	AUROC [95% CI]	p-value	Se (%)	Sp (%)
**District hospital (Tier2)**						
Overall	***1452***		0·83 [0·79–0·86]	--------	79	87
Sex	***1452***			0·462		
Female	*516*	(35·5)	0·85 [0·78–0·91]		77	92
Male	*936*	(64·5)	0·82 [0·78–0·86]		80	83
Age (years)	***1452***			0·725		
<30	*378*	(26·0)	0·82 [0·76–0·88]		79	85
≥30	*1074*	(74·0)	0·83 [0·79–0·87]		79	87
Excessive alcohol intake	***991***			0·941		
No	*872*	(88·0)	0·83 [0·79–0·88]		81	86
Yes	*119*	(12·0)	0·84 [0·74–0·93]		83	84
Region	***1452***			0·127		
West SSA	*1370*	(94·4)	0·84 [0·80–0·87]		80	87
East/South SSA	82	(5·6)	0·74 [0·63–0·86]		74	75
Hospital/community	***1452***			0·735		
Hospital-based	*777*	(53·5)	0·82 [0·78–0·87]		79	85
Community-based screening	675	(46·5)	0·83 [0·78–0·89]		79	88
**Regional hospital (Tier3-HBeAg)**						
Overall	***1439***		0·88 [0·85– 0·90]	--------	91	84
Sex	***1439***			0·215		
Female	*510*	(35·4)	0·90 [0·85–0·95]		90	90
Male	*929*	(64·6)	0·86 [0·83–0·89]		92	81
Age (years)	***1439***			0·010		
<30	*378*	(26·3)	0·82 [0·77–0·88]		85	79
≥30	*1061*	(73·7)	0·90 [0·87–0·93]		94	86
Excessive alcohol intake	***979***			0·666		
No	*863*	(88·2)	0·90 [0·87–0·92]		95	84
Yes	*116*	(11·8)	0·88 [0·81–0·95]		94	82
Region	***1439***			0·036		
West SSA	*1365*	(94·9)	0·88 [0·86–0·91]		91	85
East/South SSA	74	(5·1)	0·77 [0·68–0·87]		89	66
Hospital/community	***1452***			0·045		
Hospital-based	*764*	(53·1)	0·86 [0·82–0·89]		89	83
Community-based screening	675	(46·9)	0·91 [0·87–0·94]		95	86
**Regional hospital (Tier3-DNA)**						
Overall	***1452***		0·85 [0·82–0·88]	--------	88	82
Sex	***1452***			0·370		
Female	*516*	(35·5)	0·86 [0·80–0·93]		82	91
Male	*936*	(64·5)	0·83 [0·80–0·86]		90	76
Age (years)	***1452***			0·998		
<30	*378*	(26·0)	0·84 [0·80–0·89]		92	77
≥30	*1074*	(74·0)	0·84 [0·81–0·88]		86	83
Excessive alcohol intake	***991***			0·561		
No	*872*	(88·0)	0·88 [0·85–0·91]		92	83
Yes	*119*	(12·0)	0·85 [0·77–0·93]		89	81

Region	***1452***			0·998		
West SSA	*1370*	(94·4)	0·84 [0·81–0·87]		86	82
East/South SSA	82	(5·6)	0·84 [0·78–0·90]		100	68
Hospital/community	***1452***			0·066		
Hospital-based	*777*	(53·5)	0·82 [0·78– 0·86]		87	78
Community-based screening	675	(46·5)	0·88 [0·83–0·92]		89	86
